# Clopidogrel Plus Aspirin in Patients With Different Types of Single Small Subcortical Infarction

**DOI:** 10.3389/fneur.2021.631220

**Published:** 2021-03-29

**Authors:** Guangyao Wang, Xiaomeng Yang, Jing Jing, Xingquan Zhao, Liping Liu, Chunxue Wang, David Wang, Anxin Wang, Xia Meng, Yongjun Wang, Yilong Wang

**Affiliations:** ^1^Department of Neurology, Beijing Tiantan Hospital, Capital Medical University, Beijing, China; ^2^China National Clinical Research Center for Neurological Diseases, Beijing, China; ^3^Neurovascular Division, Department of Neurology, Barrow Neurological Institute, St. Joseph's Hospital and Medical Center, Phoenix, AZ, United States

**Keywords:** SSSI, PAD, lesion location, dual antiplatelet, prognosis

## Abstract

**Background:** We aim to investigate the effects and safety of clopidogrel plus aspirin in patients with different types of single small subcortical infarction (SSSI) in the Clopidogrel in High-risk patients with Acute Non-disabling Cerebrovascular Events (CHANCE) trial.

**Methods:** SSSI was defined as single DWI lesion of ≤2.0 cm. Patients with SSSI were divided into SSSI + PAD (parent artery disease) and SSSI – PAD, according to the stenosis of the parent artery. The efficacy outcome was stroke recurrence during 90-day follow-up. Cox proportional hazards models or logistic regression models were used to assess the interaction of the treatment effects of clopidogrel plus aspirin vs. aspirin alone among patients with and without PAD.

**Results:** Among 338 patients with SSSI included in the subanalysis, 105 were with PAD and 233 without. The efficacy of clopidogrel plus aspirin compared with aspirin alone on any stroke was consistent between patients with [adjusted hazard ratio (HR) 0.84; 95% confidence interval (CI), 0.25–2.75] and without PAD (adjusted HR 1.03; 95% CI, 0.40–2.68, interaction *P* = 0.83). In patients with SSSI + PAD, the rate of stroke recurrence in those treated with dual antiplatelet therapy and mono antiplatelet therapy was not significantly different (10.9 vs. 13.6%, *P* = 0.77). The number of bleeding events was similar between the clopidogrel-aspirin group and aspirin group regardless of SSSI + PAD or SSSI – PAD.

**Conclusions:** There was no significant difference in the efficacy of clopidogrel plus aspirin compared with aspirin alone between patients with SSSI + PAD and SSSI – PAD in the CHANCE trial. Studies in other populations and with adequate power are needed to further verify such findings.

## Introduction

Single small subcortical infarction (SSSI), commonly known as lacunar stroke, is an important ischemic stroke (IS) subtype (1–5), and accounts for 25% of all IS in westerners (6) and 16.8–42.0% in Chinese (7–9). A recent study showed that SSSI in perforator territory had a heterogeneous pathogenesis regarding the presence of parental arterial disease (PAD) and SSSI associated with PAD (SSSI + PAD) had higher prevalence of atherosclerosis than those without PAD (SSSI – PAD) (10). SSSI – PAD were probably related to fibrinoid necrosis or “lipohyalinosis” of small perforating arteries (11).

At present, little is known about the optimal antiplatelet strategy for early stroke prevention in patients with lacunar strokes. The Secondary Prevention of Small Subcortical Strokes (SPS3) trial has found that the addition of clopidogrel to aspirin did not significantly reduce the risk of recurrent stroke but significantly increased the risk of bleeding and death among patients with recent lacunar strokes (12). The second Cilostazol Stroke Prevention Study (CSPS 2) showed Cilostazol seemed not to be inferior to aspirin for the prevention of stroke after lacunar stroke (13).

Several studies have indicated that dual antiplatelet therapy with clopidogrel and aspirin was an effective treatment in patients with symptomatic carotid or intracranial arterial stenosis compared to aspirin alone (hereinafter, “mono antiplatelet therapy”) (14–16). Considering the heterogeneous pathogenesis of SSSI, whether IS patients with different types of SSSI based on the presence or absence of PAD might benefit from dual antiplatelet therapy is still uncertain.

In the Clopidogrel in High-risk patients with Acute Non-disabling Cerebrovascular Events (CHANCE) trial, clopidogrel plus aspirin reduced the risk of recurrent stroke in Chinese patients with acute minor stroke or high-risk transient ischemic attack (TIA). Therefore, we aim to investigate whether different types of SSSI (SSSI + PAD or SSSI – PAD) can benefit from dual antiplatelet therapy in this subgroup analysis of CHANCE trial.

## Methods and Materials

### Study Design

The design and main results for CHANCE trial have been described previously (9, 17). In brief, a total of 5,170 patients from 114 clinic centers within 24 h after the onset of minor ischemic stroke or high-risk TIA to combination therapy with clopidogrel and aspirin (clopidogrel at an initial dose of 300 mg, followed by 75 mg per day for 90 days, plus aspirin at a dose of 75 mg per day for the first 21 days) or to placebo plus aspirin (75 mg per day for 90 days). CHANCE was registered with ClinicalTrials.gov, identifier NCT00979589. The imaging subgroup study was approved by the ethics committees of all participating centers.

### Efficacy and Safety Outcomes

The primary outcome of the CHANCE trial was stroke (ischemic or hemorrhagic) during the 90-day follow-up in an intention-to-treat analysis. Secondary efficacy outcomes included a new clinical vascular event at 90 days (IS, hemorrhagic stroke, myocardial infarction, or vascular death)—analyzed as a composite outcome and individual outcomes as well, and disabling/fatal stroke (modified Rankin Scale score of 2 to 6 at 90 days). The primary safety outcome was a moderate-to-severe bleeding event at 90 days, as per the Global Utilization of Streptokinase and Tissue Plasminogen Activator for Occluded Coronary Arteries (GUSTO) definition (18). Severe bleeding event was defined as a fatal or intracranial hemorrhage or other hemorrhage causing hemodynamic compromise requiring treatment. Moderate bleeding event was defined as bleeding requiring blood transfusion. Other safety outcomes included mild bleeding by the GUSTO definition and any bleeding event.

### Subjects

Of 5,170 patients enrolled in CHANCE, 1,089 consecutive patients participated in the imaging subgroup study. All MRI/MRAs of the brain were performed within 7 days of symptom onset. Patients with SSSI were selected for this analysis. SSSI was defined as a single DWI lesion of ≤2.0 cm in size at its largest dimension in the perforator territory of the middle cerebral artery and basilar artery (infarctions in the paramedian pontine area) (10), SSSI with stenosis of any degree of the parent artery (middle cerebral artery and basilar artery) was regarded as a SSSI + PAD and SSSI without stenosis of the parent artery as SSSI – PAD.

### Image Analysis/Interpretation

**Figure 1:**
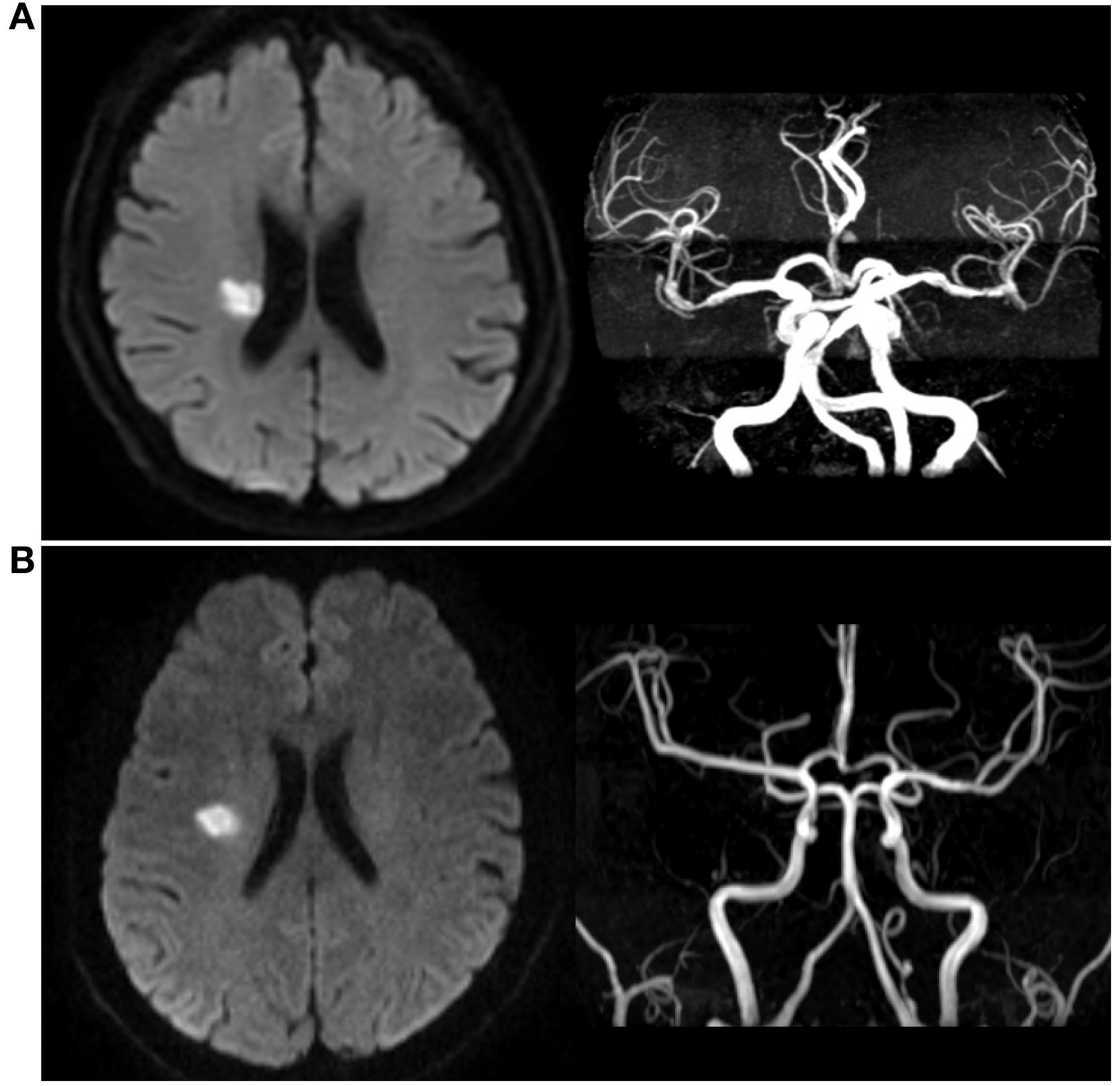
Different types of single small subcortical infarction. **(A)** Single small subcortical infarction associated with parental artery disease (SSSI + PAD); **(B)** Single small subcortical infarction without parental artery disease (SSSI – PAD).

The method of the imaging subgroup analysis has been described before (19, 20). Briefly, all patients in the imaging subgroup study of the CHANCE trial underwent conventional MRI of brain and three-dimensional (3D) time-of-flight magnetic resonance angiography (MRA) with a 3.0 or 1.5 T MR scanner. Other MR sequences included T1/T2-weighted imaging and diffusion-weighted imaging (DWI). All MRI/MRA images were stored in digital format and read centrally by two readers who were blinded to the subjects' clinical information or outcomes. In cases of discrepancy, the final diagnosis was reached by consensus. We assessed the following arterial segments: middle cerebral artery (M1/M2) and basilar artery, degree of intracranial stenosis on MRA was calculated by using the published method described in the Warfarin-Aspirin Symptomatic Intracranial Disease Study (21). The status of the artery was categorized as normal or PAD. Absence of distal filling on MRA would be regarded as occlusion. In this study, stenosis of any degree of PAD was regarded as a significant cause of SSSI as described in previous study (10). Patients with SSSI were divided into SSSI + PAD or SSSI – PAD groups (Figure 1).

### Statistical Analysis

We compared the baseline characteristics of patients with SSSI + PAD or SSSI – PAD by using chi-square tests and independent sample *t* tests for categorical and continuous variables, respectively. In addition, the baseline characteristics of patients with SSSI + PAD or SSSI-PAD on the dual antiplatelet therapy or mono antiplatelet therapy were also compared. The rates of primary and secondary efficacy and safety outcomes at 90 days were compared between patients on different treatments (dual antiplatelet therapy, or mono antiplatelet therapy) with SSSI + PAD or SSSI – PAD by using chi-square tests.

Cox proportional hazards models or logistic regression models were performed with different treatments as the covariates, to obtain the hazard ratios (HR) or odds ratios (OR) and two-sided 95% confidence intervals (CI) of different treatments for the primary efficacy outcome of any stroke and the safety outcome of any bleeding, regardless of the presence of PAD. In the model, we had adjusted for age, sex, systolic blood pressure, previous history of ischemic stroke, previous history of smoking, and time to randomization. Cox proportional hazards models or logistic regression models were also performed with the treatments (clopidogrel plus aspirin or placebo plus aspirin), the presence of PAD, and the treatment by presence of PAD interaction as covariates, to test the interaction between the differential effects of dual vs. mono antiplatelet therapies on the primary efficacy outcome (any stroke) and safety outcomes among patients with and without PAD. The time to the primary efficacy outcome event for each group was presented by the Kaplan-Meier curves. All tests were two-sided, and a *P*-value <0.05 was considered to be statistically significant. All statistical analyses were performed with the use of SAS software, version 9.0 (SAS Institute).

## Results

Between October 2009 and July 2012, a total of 5,170 patients with acute minor stroke or high-risk TIA were enrolled in the CHANCE trial. Of those, 1,089 patients at 45 centers undergoing MR examinations at baseline with all the sequences as required were included in this subgroup analysis. Compared to patients not in the imaging subgroup study, patients in the imaging subgroup study were older, more likely to have higher systolic blood pressure, lower body mass index, longer time to be randomized, higher baseline ABCD2 score for qualifying TIA, minor stroke as the qualifying event and less likely to have prior history of IS (Supplementary Table 1).

In the imaging subgroup, 338 with SSSI were recruited in the final analysis of this study. Among them 105 patients had SSSI + PAD and 233 had SSSI – PAD. Patients with SSSI + PAD were older (64.3 vs. 59.6, *P* < 0.001) and more likely to be female (47.6 vs. 30.0%, *P* = 0.002). They had higher systolic blood pressure (163.2 vs. 156.4, *P* = 0.04), as compared with those with SSSI – PAD (Table 1). In patients with SSSI + PAD, more in the dual antiplatelet therapy group had a prior history of ischemic stroke (28.3 vs. 11.9%, *P* = 0.05) and were smokers (43.5 vs. 20.3%, *P* = 0.02), as compared to those in the mono antiplatelet therapy group (Table 1). In patients with SSSI – PAD, patients in the dual antiplatelet therapy group were older (61.5 vs. 57.5, *P* = 0.003), shorter time to be randomized (13.3 vs. 15.2, *P* = 0.02) than those in the mono antiplatelet therapy group (Table 1). Other baseline characteristics were not significantly different between the two groups.

**Table 1:** Baseline characteristics of the patients with SSSI + PAD and SSSI – PAD.

**Characteristics**	**SSSI+PAD**			**SSSI – PAD**			
	**Dual antiplatelet,** ***n* (%)**	**Mono antiplatelet,** ***n* (%)**	***P-* Value**	**Dual antiplatelet,** ***n* (%)**	**Mono antiplatelet,** ***n* (%)**	***P-* Value**	*P-* Value^*^
Patients	46 (43.8)	59 (56.2)		124 (53.2)	109 (46.9)		
Age(years)	63.6 ± 9.4	64.8 ± 9.8	0.46	61.5 ± 10.1	57.5 ± 10.1	0.003	<0.001
Male	26 (56.5)	29 (49.2)	0.56	80 (64.5)	83 (76.1)	0.06	0.002
Systolic blood pressure(mmHg)	157.8 ± 24.5	167.4 ± 27.3	0.12	157.1 ± 21.4	155.6 ± 22.8	0.54	0.04
Diastolic blood pressure (mmHg)	91.4 ± 14.0	92.7 ± 12.7	0.33	91.1 ± 13.6	92.7 ± 14.2	0.40	1.00
Body Mass Index (kg/m^2^)	25.1 ± 2.8	24.4 ± 3.2	0.11	24.3 ± 3.1	24.7 ± 3.2	0.36	0.33
Previous history
Ischemic stroke	13 (28.3)	7 (11.9)	0.05	15 (12.1)	15 (13.8)	0.84	0.14
TIA	0	2 (3.4)	0.50	1 (0.8)	1 (0.9)	1.00	0.59
Myocardial infarction	1 (2.2)	0	0.44	4 (3.2)	1 (0.9)	0.37	0.67
Angina	0	2 (3.4)	0.50	3 (2.4)	0	0.25	0.65
Cardiac dysfunction	2 (4.3)	0	0.19	1 (0.8)	0	1.00	0.23
Arrhythmia	2 (4.3)	0	0.19	1 (0.8)	3 (2.8)	0.34	1.00
Valvular heart disease	0	0	NA	0	1 (0.9)	0.47	1.00
Hypertension	33 (71.7)	42 (71.2)	1.00	72 (58.1)	69 (63.3)	0.42	0.07
Diabetes mellitus	13 (28.3)	12 (20.3)	0.37	25 (20.2)	15 (13.8)	0.23	0.18
Hyperlipidemia	5 (10.9)	6 (10.2)	1.00	15 (12.1)	9 (8.3)	0.39	1.00
Smoking	20 (43.5)	12 (20.3)	0.02	57 (46.0)	58 (53.2)	0.30	0.001
Time to randomization (hours)	12.4 ± 7.0	13.1 ± 6.5	0.61	13.3 ± 6.4	15.2 ± 6.5	0.02	0.07
Time to randomization							
<12 h	25 (54.3)	28 (47.5)	0.56	58 (46.8)	38 (34.9)	0.08	0.12
Medications							
Antihypertensive	22 (50.0)	25 (42.4)	0.55	56 (45.9)	50 (46.3)	1.00	1.00
Antidiabetic	8 (18.2)	9 (15.3)	0.79	19 (15.6)	11 (10.2)	0.25	0.40
Lipid-lowering	19 (43.2)	31 (52.5)	0.43	68 (55.7)	61 (56.5)	1.00	0.23

*SSSI + PAD, Single small subcortical infarction with parental arterial disease; SSSI – PAD, Single small subcortical infarction without parental arterial disease; TIA, Transient Ischemic Attack*.

* *P-values for comparisons between patients with and without PAD*.

### Efficacy Outcomes

**Figure 2:**
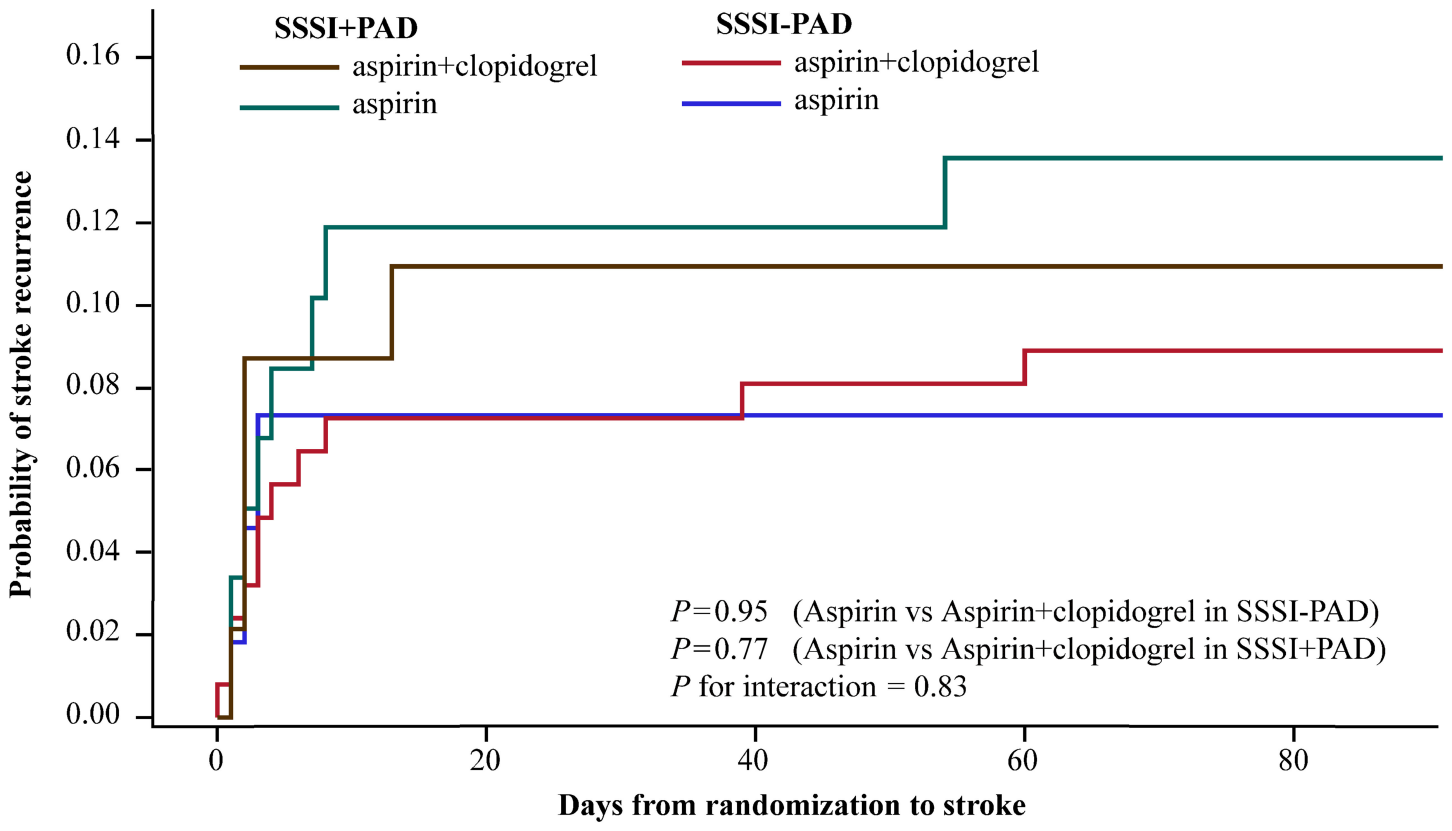
Kaplan-Meier curves for the primary efficacy outcome of any stroke. Kaplan-Meier curves showing the time to the primary efficacy outcome event (any stroke) in patients with SSSI + PAD and SSSI – PAD, treated with clopidogrel plus aspirin, or placebo plus aspirin. SSSI, single small subcortical infarction; PAD, parental arterial disease.

In our study, 32 of the 338 patients (9.5%) with SSSI had a primary efficacy outcome of recurrent stroke during the 90-day follow-up period (Table 2). Figure 2 shows the Kaplan-Meier curves presenting the time to event for the primary efficacy outcome in different groups. The addition of clopidogrel to aspirin did not significantly reduce stroke recurrence than aspirin alone among patients with SSSI + PAD (adjusted HR 0.84; 95% CI, 0.25–2.75; *P* = 0.77) and those with SSSI – PAD (adjusted HR 1.03; 95% CI, 0.40–2.68; *P* = 0.95) (interaction *P* = 0.83; Table 2). In patients with SSSI + PAD, the rate of stroke recurrence in those treated with dual antiplatelet therapy and mono antiplatelet therapy was not significantly different (10.9 vs. 13.6%, *P* = 0.77) (Table 2). Dual antiplatelet therapy did not reduce stroke recurrence in all SSSI patients (Supplementary Table 2).

**Table 2:** Efficacy and safety outcomes of the patients with SSSI + PAD and SSSI – PAD.

	**SSSI + PAD**				**SSSI – PAD**				
**Outcomes**	**Mono antiplatelet,** ***n* (%)**	**Dual antiplatelet,** ***n* (%)**	**HR/OR** (95% CI) ^*^	*P-*Value^*^	**Mono antiplatelet,** ***n* (%)**	**Dual antiplatelet,** ***n* (%)**	**HR/OR** (95% CI)^*^	*P*-Value^*^	***P*-Value^‡^**
Efficacy outcomes									
Primary efficacy outcome, stroke	8 (13.6)	5 (10.9)	0.84 (0.25–2.75)	0.77	8 (7.3)	11 (8.9)	1.03 (0.40–2.68)	0.95	0.83
Secondary efficacy Outcome^†^									
Ischemic stroke	8 (13.6)	5 (10.9)	0.84 (0.25–2.75)	0.77	8 (7.3)	11 (8.9)	1.03 (0.40–2.68)	0.95	0.83
Hemorrhagic stroke	0	0	NA	NA	0	0	NA	NA	NA
Myocardial infarction	0	0	NA	NA	0	0	NA	NA	NA
Vascular death	0	0	NA	NA	0	0	NA	NA	NA
Death from any cause	0	0	NA	NA	0	0	NA	NA	NA
TIA	1 (1.7)	0	NA	NA	2 (1.8)	0	NA	NA	NA
Disabling/fatal stroke	9 (15.3)	7 (15.9)	1.21 (0.37–3.97)	0.75	9 (8.3)	11 (9.0)	1.02 (0.39–2.72)	0.96	0.96
Safety outcomes									
Bleeding, according to GUSTO									
Severe Bleeding	0	1 (2.2)	NA	NA	0	1 (0.8)	NA	NA	0.99
Moderate Bleeding	0	0	NA	NA	0	0	NA	NA	NA
Mild Bleeding	0	0	NA	NA	0	0	NA	NA	NA
Any bleeding	1 (1.7)	3 (6.5)	4.00 (0.34–46.82)	0.27	3 (2.8)	2 (1.6)	0.33 (0.05–2.24)	0.25	0.13

*CI, confidence interval; HR, hazard ratio; OR, odds ratio; GUSTO, Global Utilization of Streptokinase and Tissue Plasminogen Activator for Occluded Coronary Arteries criteria; SSSI + PAD, Single small subcortical infarction with parental arterial disease; SSSI – PAD, Single small subcortical infarction without parental arterial disease*.

* *Adjusted for age, male, systolic blood pressure, previous history of ischemic stroke, smoking and time to randomization*.

† *Secondary efficacy outcome: new clinical vascular events including ischemic stroke, hemorrhagic stroke, myocardial infarction, or vascular death*.

‡ *P-values for interaction of treatment by presence of PAD*.

### Safety Outcomes

Two patients in the dual therapy had severe bleeding events: one in SSSI + PAD group and one in SSSI – PAD group. The adjusted HR for the clopidogrel plus aspirin vs. aspirin alone on any bleeding event was 4.00 (95% CI, 0.34–46.82) in patients with SSSI + PAD and 0.33 (95% CI, 0.05–2.24) in patients with SSSI – PAD, respectively. No statistically significant evidence for the interaction between the types of SSSI and treatment allocation on any bleeding event (interaction *P* = 0.16; Table 2). Dual antiplatelet therapy did not increase the risk of any bleeding event in all SSSI patients (Supplementary Table 2).

## Discussion

We found that the combination of clopidogrel with aspirin did not reduce stroke recurrence in patients with SSSI regardless of PAD in the CHANCE trial. To our knowledge, the current subgroup analysis was the first to explore the efficiency of short-term (21 days) dual antiplatelet therapy in patients with different types of SSSI.

A few clinical trials had assessed the role of combining clopidogrel and aspirin for non-cardioembolic IS prevention (12, 14, 15, 22). The SPS3 trial was similar to this analysis, and it has concluded that dual antiplatelet therapy did not significantly reduce the risk of recurrent stroke but did significantly increase the risk of bleeding and death among patients with recent small subcortical infarctions compared to those on mono antiplatelet therapy (12). Similarly, our subgroup analysis indicated that dual antiplatelet therapy did not significantly reduce the risk of recurrent stroke in those with small subcortical infarctions. As for the risk of hemorrhage, unlike our result, the SPS3 trial found that the risk of major hemorrhage was almost doubled among those on dual antiplatelet therapy. One possible explanation would be the duration of dual antiplatelet therapy. Patients in the CHANCE trial were on dual antiplatelet therapy for 21 days while the mean duration of such treatment was 3.4 years in the SPS3 trial. Previous studies have showed that the risk of bleeding was low if the treatment was within 21 days, but increased if treated long-term (23–25). Other studies also indicated that short-term (7 days) dual antiplatelet therapy did not increase the risk of hemorrhage in patients with large artery atherosclerotic IS (14–16).

SSSI in penetrating arterial territory could be caused by the plaque from the parental artery blocking the orifice of penetrators (SSSI + PAD) or lipohyalinosis of distal small arteries (26–29). Therefore, SSSI + PAD was classified as large artery atherosclerosis instead of small artery disease in a new classification system of ischemic stroke (5). Previous trials have indicated that dual antiplatelet therapy could reduce microembolic signals in patients with predominantly intracranial symptomatic arterial stenosis (15) or carotid stenosis (14). Results from these trials supported the hypothesis that dual antiplatelet therapy was effective in treating large artery atherosclerosis stroke. In spite of the possibility of a greater effect in SSSI + PAD patients with dual antiplatelet therapy, non-significant difference was observed in our study. The fact that patients with SSSI + PAD in our study did not have significantly more indicators of atherosclerosis than those with SSSI – PAD may be one underlying cause. In addition, the number of patients in this subgroup analysis probably was underpowered to detect any significant difference between the effects of dual vs. mono-antiplatelet therapies.

There were several limitations in our study. First, only approximately 20.0% of patients in the CHANCE trial were analyzed in imaging subgroup analysis, and there were small numbers of outcome events, especially for the safety outcomes of bleeding events. It might indicate potential selection bias of the current study, considering the fact that we only included cases from 45 of 114 participating centers providing MRIs. Therefore, the current study had limited power to detect heterogeneities of the efficiency and safety of dual vs. mono antiplatelet therapies among patients with and without PAD. Secondly, all patients with SSSI in the CHANCE trial had minor stroke (National Institute of Health stroke scale score ≤3), so the extrapolation of findings from CHANCE to other populations should be made with caution. Third, we could not completely rule out the possibility that MRA lesions were due to a partial embolic occlusion as we did not exclude patients with stenosis of the ipsilateral carotid artery or vertebral artery. However, the possibility of embolic occlusion should be relatively low in our study considering embolic MCA occlusion rarely causes SSSI if infarcts are assessed with DWI according to previous studies (27, 30), and extracranial large-artery stenosis is less common in Chinese patients (31). Fourth, in light of the small sample size in this analysis, patients with SSSI + PAD were not additionally classified by the degree of artery stenosis. Future large-scale studies are needed.

## Conclusion

Our results support the hypothesis that dual antiplatelet therapy, initiated early after ictus and lasting for a short period, does not reduce the risk of any stroke among patients with SSSI regardless of PAD. Studies on other populations with large sample size and implying HRMRI are needed in the future to verify our findings further in patients with different types of SSSI.

## Data Availability Statement

The raw data supporting the conclusions of this article will be made available by the authors, without undue reservation.

## Ethics Statement

The studies involving human participants were reviewed and approved by The Ethics Committee of Beijing Tiantan Hospital. The patients/participants provided their written informed consent to participate in this study.

## Author Contributions

GW and XY interpreted the data and drafted the manuscript. JJ, XZ, LL, CW, and XM acquired the data and revised the manuscript. AW analyzed the data. DW revised the manuscript. YoW and YiW designed the research and handled funding and supervision. All authors have read and agreed on the final manuscript.

## Conflict of Interest

The authors declare that the research was conducted in the absence of any commercial or financial relationships that could be construed as a potential conflict of interest.

## Abbreviations

SSSI: single small subcortical infarction
PAD: parent artery disease
CHANCE: Clopidogrel in High-risk patients with Acute Non-disabling Cerebrovascular Events
IS: ischemic stroke
SPS3: Secondary Prevention of Small Subcortical Strokes
TIA: transient ischemic attack
GUSTO: Global Utilization of Streptokinase and Tissue Plasminogen Activator for Occluded Coronary Arteries
DWI: diffusion-weighted imaging
HR: hazard ratio
CI: confidence interval.
