# Supplementary material for: Clopidogrel Plus Aspirin in Patients With Different Types of Single Small Subcortical Infarction
Source: Front Neurol. 2021 Mar 29;12:631220. doi: 10.3389/fneur.2021.631220 (PMC8039512; doi:10.3389/fneur.2021.631220)
Supplement: Supplementary file 1 [file Table_1.pdf]

Supplementary Table 1 Baseline characteristics of patients in the CHANCE trial included in the current subgroup analysis or not.

Characteristics	Not included (n=4,081)	Included (n=1,089)	*P* value
Age (years)	62.0 (54.6-71.2)	63.1 (55.2-71.6)	0.048
Women	1374 (33.7)	376 (34.5)	0.614
Systolic blood pressure (mmHg)	150.0 (135.0-160.0)	150.0 (140.0-167.0)	<0.001
Diastolic blood pressure (mmHg)	90.0 (80.0-100.0)	90.0 (80.0-100.0)	0.073
Body mass index (kg/m^2)	24.5 (22.9-26.6)	24.3 (22.5-26.2)	0.001
Medical history			
Ischaemic stroke	846 (20.7)	187 (17.2)	0.009
TIA	142 (3.5)	32 (2.9)	0.449
Myocardial infarction	77 (1.9)	19 (1.7)	0.899
Angina	155 (3.8)	29 (2.7)	0.080
Congestive heart failure	61 (1.5)	19 (1.7)	0.580
Known atrial fibrillation or flutter	75 (1.8)	21 (1.9)	0.802
Valvular heart disease	10 (0.2)	4 (0.4)	0.511
Hypertension	2689 (65.9)	710 (65.2)	0.667
Diabetes mellitus	866 (21.2)	227 (20.8)	0.802
Hypercholesterolaemia	436 (10.7)	137 (12.6)	0.082
Pulmonary Embolism	1 (0.02)	0	1.000
Current or previous smoking	1763 (43.2)	458 (42.1)	0.513
Time to randomization (hours)	12.0 (6.1-19.5)	12.0(7.2-19.5)	0.013
Time to randomization, dichotomized			0.811
< 12 hours	2035 (49.9)	538 (49.4)	
>= 12h hours	2046 (50.1)	551 (50.6)	
Qualifying event			0.003
TIA	1180 (28.9)	265 (24.3)	
Minor stroke	2901 (71.1)	824 (75.7)	
Baseline $ABCD^2$ score for qualifying TIA*	4 (4-5)	5 (4-5)	0.013

Data are median (IQR) or number (%).
Statistical analyses were performed by Wilcoxon rank sum tests or chi-square tests.
*Only for patients recruited to the CHANCE trial with TIA as a qualifying event (n=1,445).
CHANCE trial=the Clopidogrel in High-risk patients with Acute Non-disabling Cerebrovascular Events trial. ICAS=intracranial arterial stenosis. TIA=transient ischaemic attack. $ABCD^2$ score=a score to predict stroke risk early after a transient ischaemic attack; acronyms: A for age; B, blood pressure; C, clinical features; D2, duration of TIA, and presence of diabetes.

Supplementary Table 2 Efficacy and safety outcomes of the patients with SSSI.

Outcomes	Mono antiplatelet, n (%)	Dual antiplatelet, n (%)	HR/OR (95% CI) *	*P* Value*
Efficacy outcomes				
Primary efficacy outcome, stroke	16(9.5)	16(9.4)	0.89(0.44-1.81)	0.76
Secondary efficacy Outcome†				
Ischemic stroke	16(9.5)	16(9.4)	0.89(0.44-1.81)	0.76
Hemorrhagic stroke	0	0	NA	NA
Myocardial infarction	0	0	NA	NA
Vascular death	0	0	NA	NA
Death from any cause	0	0	NA	NA
TIA	3(1.8)	0(0)	NA	1.00
Disabling/fatal stroke	18(10.8)	18(10.8)	0.93(0.46-1.88)	0.83
Safety outcome				
Bleeding, according to GUSTO				
Severe Bleeding	0	2(1.2)	NA	1.00
Moderate Bleeding	0	0	NA	NA
Mild Bleeding	0	0	NA	NA
Any bleeding	4(2.4)	5(2.9)	1.13(0.29-4.35)	0.86

Abbreviations: CI=confidence interval; HR=hazard ratio; OR=odds ratio; GUSTO= Global Utilization of Streptokinase and Tissue Plasminogen Activator for Occluded Coronary Arteries criteria; SSSI = Single small subcortical infarction.

*Adjusted for age, male, systolic blood pressure, previous history of ischemic stroke, smoking and time to randomization.

†Secondary efficacy outcome: new clinical vascular events including ischemic stroke, hemorrhagic stroke, myocardial infarction, or vascular death.
